# Supplementary material for: Quantifying protein densities on cell membranes using super-resolution optical fluctuation imaging
Source: Nat Commun. 2017 Nov 23;8:1731. doi: 10.1038/s41467-017-01857-x (PMC5700985; doi:10.1038/s41467-017-01857-x)
Supplement: Supplementary file 1 — Supplementary Information [file 41467_2017_1857_MOESM1_ESM.pdf]

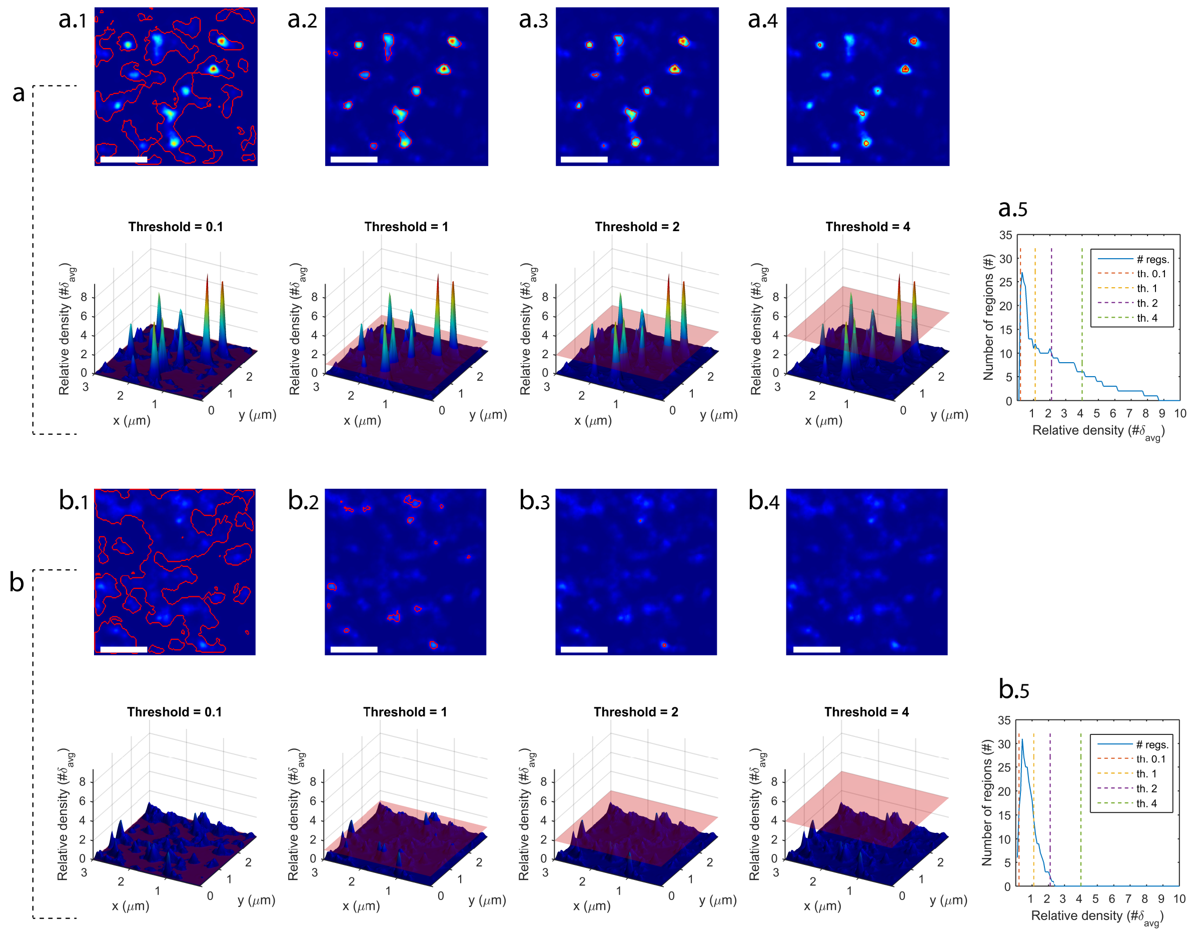

Supplementary Figure 1:

SOFI-based molecular density analysis threshold filtering (simulations).

Threshold filtering of simulated datasets containing (a) high density regions (HDRs), (b) randomly distributed emitters. First a mean density over the wall region of interests (ROIs) is calculated as $\delta_{\text{avg}} = \frac{1}{KLN}\sum_{k=1}^{K}\sum_{l=1}^{N}\sum_{n=1}^{N} d(k,l,n)$, where $d(k,l,n)$ is a molecular density per pixel located in the k-th row and l-th column of n-th ROI, N is the total number of ROIs, k,l runs through all rows and columns of the ROI, respectively. The threshold parameter is given as a multiple of the mean density taken over the selected ROI. i.e. $\textit{threshold} = 2$ corresponds to $2\delta_{\text{avg}}$. For each threshold setting, densities above the threshold determine the boundary of the density dependent area providing number of segments, area size and equivalent diameter. (a.1 - a.4) shows an example for threshold values: 0.1, 1, 2, and 4. Repeating this procedure step by step for the whole range of thesholds, we obtain charts that show number of HDRs as a function of the density threshold (a.5,b.5; blue line) for the case with HDRs (a.5) and with randomly distributed emitters (b.5). For the case with clustered emitters, 6 HDRs are detected at the density threshold 4 (a.5; green dashed line). For the case with randomly distributed emitters, 0 HDRs is detected at this density threshold (b.5; green dashed line). Scale bars: 1 μm.

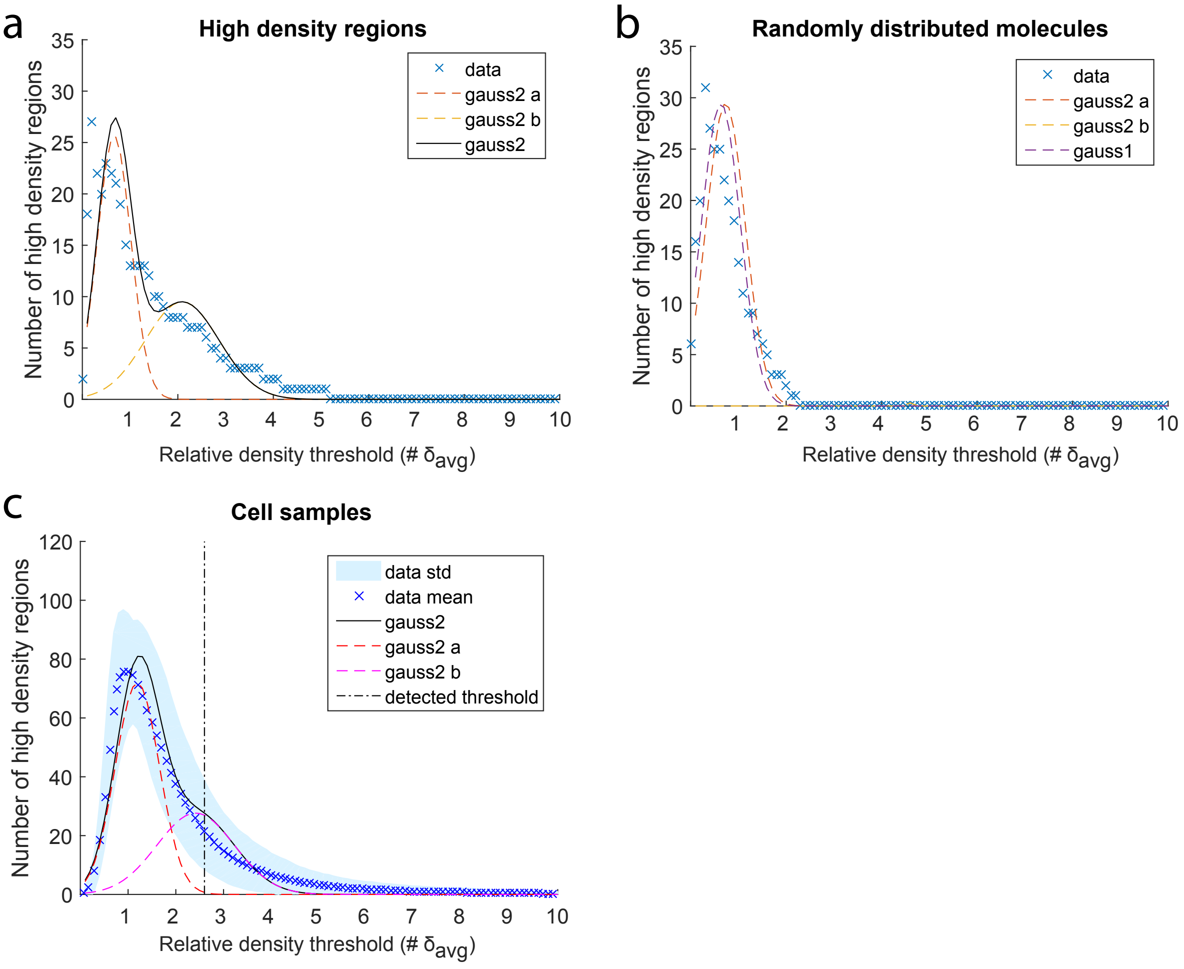

Supplementary Figure 2:

SOFI-based molecular density analysis threshold detection.

Number of high density regions (HDRs) as a function of density threshold for a simulated sample which (a) contains high density regions, or (b) contains randomly distributed molecules. Fitting a sum of two Gaussian functions reveals a component which corresponds to the random patterns (red dashed line) and a component corresponding to non random HDRs (yellow dashed line). (c) Number of HDRs as a function of density threshold averaged over all cell samples (i.e. 80 samples). Averaging across all samples allows us to obtain one density threshold for all samples and thus compare HDRs size of different CD4 variants at the same density level (Fig. 3). A sum of two Gaussian functions fitted to the data ("gauss2"). Vertical dash-dot line indicates the detected threshold where the value of the Gaussian function (red dashed line), which corresponds to randomly distributed molecules, falls below 1.

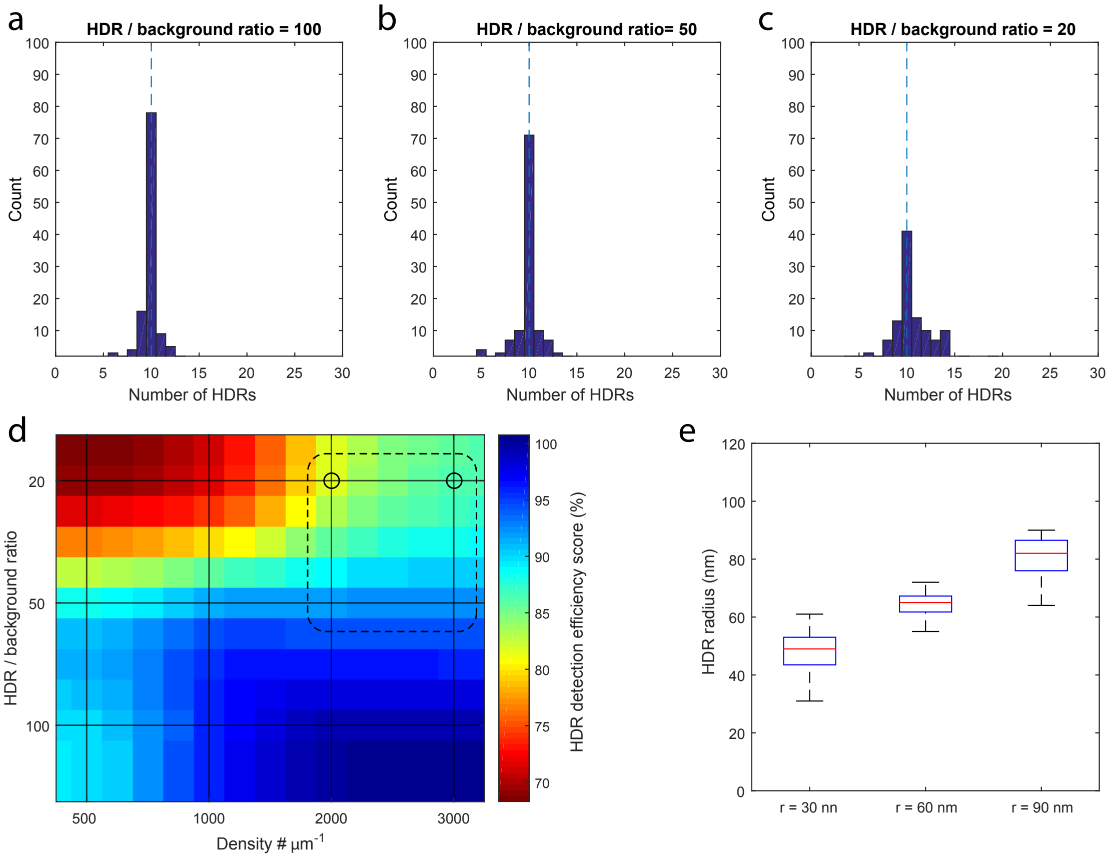

Supplementary Figure 3:

Simulation of SOFI-based molecular density analysis under controlled conditions.

Estimation of number of high density regions (HDRs): 10 simulated HDR per ROI as ground truth. The HDR radius was in the range $\{30, 60, 90\}$ nm, the molecular density per cluster was in the range $\{500, 1000, 2000, 3000\}$ molecules per μm^2. In between the HDRs, molecules were randomly distributed such that HDR/background ratio was equal to $\{100, 50, 20\}$. Each test scenario was repeated 10 times. In total, 360 datasets were generated and evaluated (120 datasets for each HDR/background case). The number of photons per emitter per frame was set to 100, which corresponds well to the experimental conditions. The number of frames of each image sequence was 5000. Dashed blue line in (a),(b),(c) marks the ground truth. Overall, the simulation validates the algorithm and estimation under a broad range of conditions. (d) HDR detection efficiency score is a probability that the estimated number of HDRs is in the range (7-13). The accuracy of the estimation increases with increasing HDR/background ratio and increasing HDR density. The simulations were calculated for the grid points. The dashed line marks the conditions where the signal to noise ratio (SNR) and signal to background ratio (SBR) of simulated data correspond to SNR and SBR of the real cell datasets in our experiments. (e) Estimation of HDR radius for grid points marked in **d** by the circles i.e. test cases with HDR/background ratio = 20, density = $\{2000, 3000\}$ molecules per μm^2. In the box plot, the box represents the interquartile range (IQR), the central mark is the median, and the whiskers extend to the most extreme data points. Each box plot was calculated over 20 measured test cases.

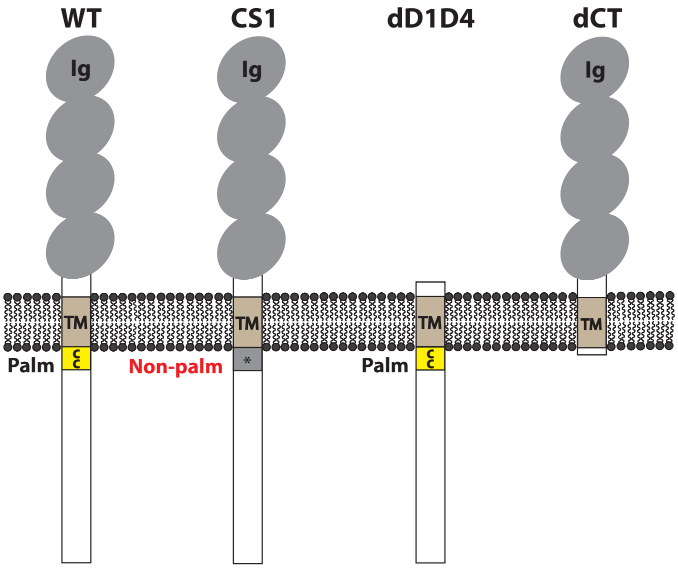

Supplementary Figure 4: Schematic representation of wild-type (WT) CD4 and its variants. Palmitoylation mutant (CS1), mutant lacking the extracellular domain (dD1D4; $CD4_{1-419}$) and mutant lacking the cytoplasmic tail (dCT; $CD4_{387-458}$). dCT mutant has a single cysteine residue (C419) in the cytosolic end of transmembrane domain which is responsible for its palmitoylation. TM = transmembrane domain, Ig = four immunoglobulin type domains D1-D4, Palm = palmitoylations sites C419 and C422, Non-palm = non-palmitoylated variant with mutations C419S, C422S. Proportions are not in scale.

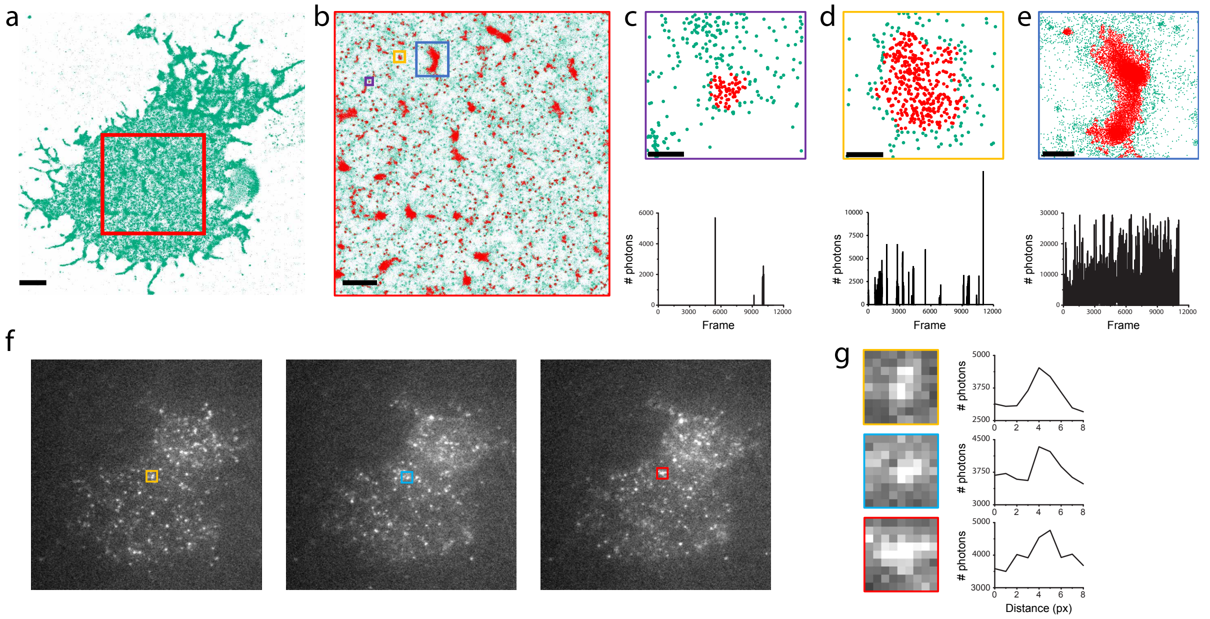

Supplementary Figure 5:

SMLM analysis of the plasma membrane organisation of wild-type CD4.

Plasma membrane organisation of wild-type CD4 protein in resting T cells characterized using photo-activation localisation microscopy (PALM) followed by a Voronoï-based segmentation algorithm [1]. High density regions (HDRs) of irregular shape frequently forming networks of connected areas were identified. Yet, since the acquisition exhibited a high density of molecules with high blinking rates, the quantification of these HDRs can be affected by localization errors and under- or overcounting artifacts as described by Burgert et al. [2]. (a) Original dataset composed of 1,747,681 localizations, scale bar 2 μm. (b) Magnification of the central area of the cell. Segmented HDRs are displayed in red, scale bar 1 μm. (c) Zoom on a HDR composed of 65 localizations, scale bar 50 nm. As shown by its time trace, the localizations in this region are originating from a single fluorophore, making its blinking correction simple. (d-e) Zoom on a denser HDR (d, 384 localizations, scale bar 50 nm) and interconnected HDRs (e, 12,886 localizations, scale bar 200 nm) with their time trace. Even for a small HDR, the presence of multiple emitters complicates the blinking correction. The problem becomes even more apparent with interconnected HDRs because of their high-density of molecules. (f-g) Three frames of the original image acquisition, pixel size 105 nm. (g) Corresponding zoom to the region covered by one HDR. The high-density of molecules makes it difficult to properly separate each emitter, resulting in localization errors as well as under-counting.

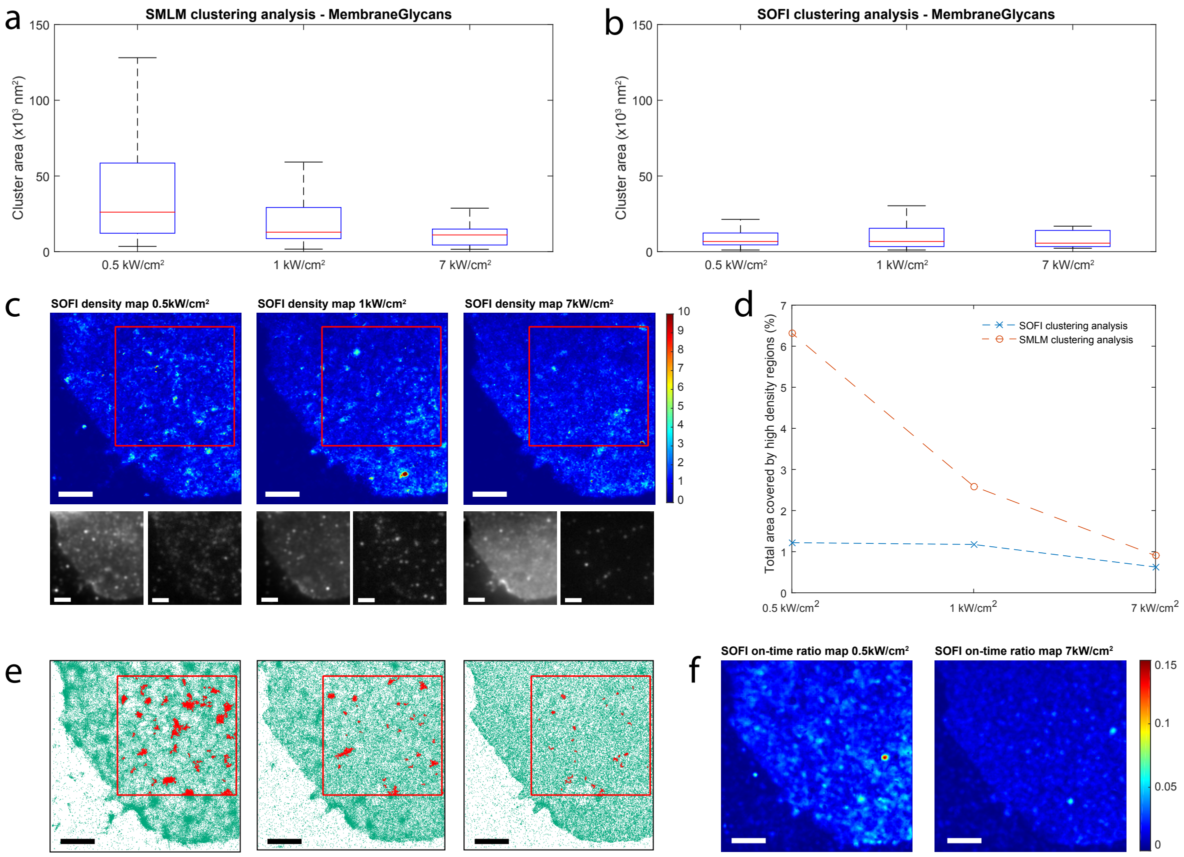

Supplementary Figure 6:

Robustness of SOFI-based clustering analysis under diverse experimental conditions.

The performance of SOFI-based clustering analysis was compared to the state-of-the-art SMLM-based clustering analysis (SR-Tesseler [3]) using samples acquired at different irradiation intensities (0.5, 1, and 7 kWcm^{-2}). For an unbiased comparison, this test was performed by evaluation of previously published data achieved by metabolic labelling of glycans on the basal membrane of U2OS cells by click chemistry and a Cy5 fluorophore-alkyne (see reference [2] for more details on sample preparation and image acquisition). High irradiation density (7 kWcm^{-2}) represents favorable conditions for SMLM. Under these conditions, the results of SMLM- and SOFI-based clustering analyses are in a good agreement. Low irradiation intensity leads to inappropriate photoswitching rates. Consequently, the input image sequence exhibits high molecular density per frame and the resulting super-resolution images show artificial membrane clusters. Even with blinking correction and the state-of-the-art SMLM-based clustering analysis [3], the data demonstrate overestimation of clusters (areas, numbers) in samples with randomly distributed molecules [2]. The box plots with SMLM-based results (a) show increasing cluster area with decreasing irradiation intensity, while no such dependence was observed when SOFI-based clustering analysis was applied (b). Cluster size and total area covered by clusters (d) remain comparable for samples irradiated by various intensities and analyzed by our SOFI based algorithm. In each box plot, the box represents the interquartile range (IQR), the central mark is the median, and the whiskers extend to the most extreme data points. Correctly, SOFI density maps (c) of all tested samples show largely homogeneous distribution of molecules. On the contrary, SMLM maps (e) contain large clusters in samples with suboptimal irradiation intensity (0.5 and 1 kWcm^{-2}). On-time ratio maps (f) reveal that lower irradiation intensity leads to higher photoswitching rates (see Supplementary Note 1 for more details about the on-time ratio). The grayscale images in the part (c) in the lower panel represent an average intensity (left panels) and a single representative frame (right panels) from the input sequence used for image and clustering analyses (ROIs). Colorbar represents relative density ($\#\delta_{\text{avg}}$; see Supplementary Figure 1). Scale bars: 2 μm. Of note, similar results were acquired by evaluation of the data from U2OS cells labelled with Alexa Fluor 647-conjugated wheat germ agglutinin which binds to glycosylated structures on the cell surface (images not shown).

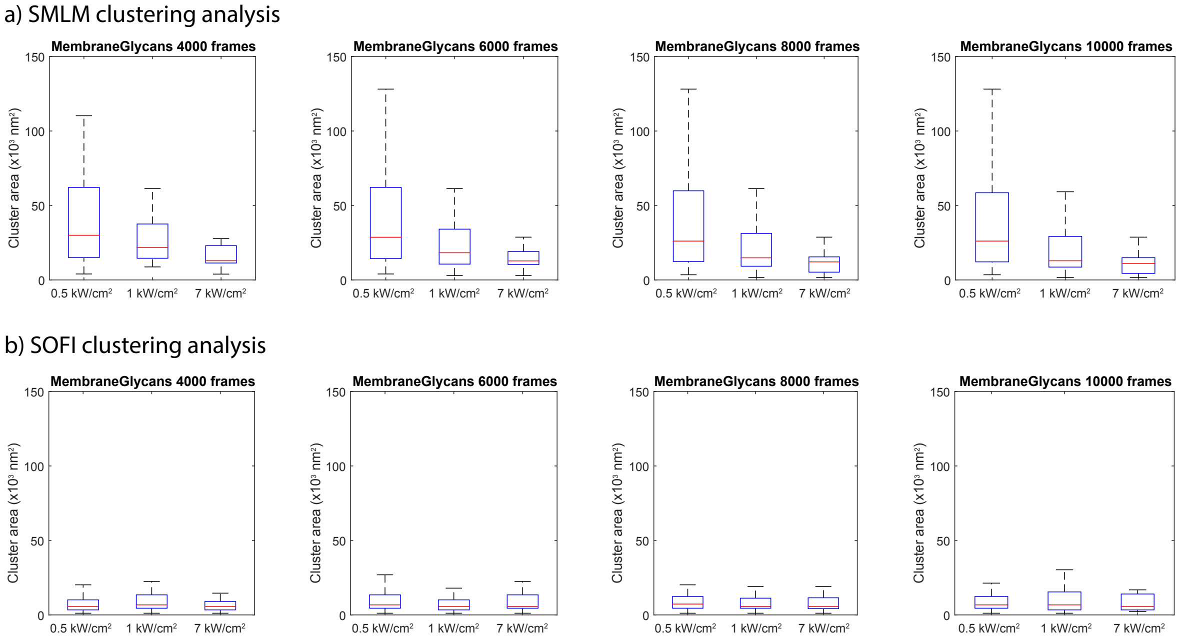

Supplementary Figure 7:

Independence of SOFI-based clustering analysis on the number of input frames.

The same data as in the Supplementary Figure 6 were analyzed using 4000, 6000, 8000 and 10000 input frames (first n-frames of the sequence) for (a) SMLM- and (b) SOFI-based clustering analysis. SOFI results (lower panel) exhibit little or no variation with varying number of input frames. Increasing number of input frames does not influence the biased results of SMLM-based clustering analysis of data acquired under suboptimal irradiation conditions (0.5 and 1 kWcm^{-2}; upper panel). In each box plot, the box represents the interquartile range (IQR), the central mark is the median, and the whiskers extend to the most extreme data points.

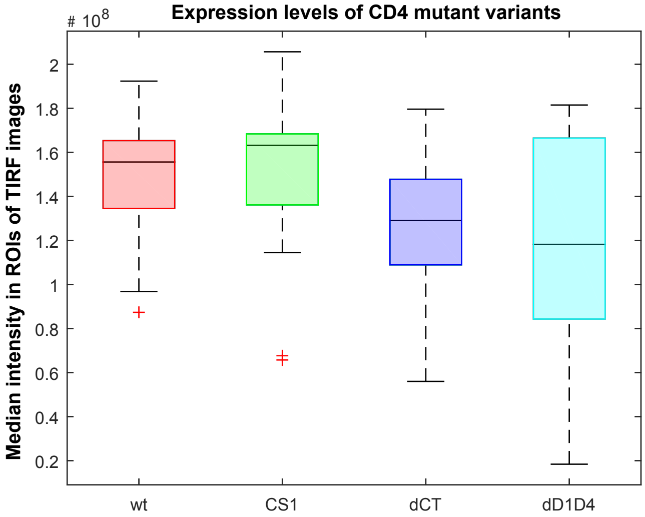

Supplementary Figure 8: Comparable expression level of CD4 variants in transfected Jurkat T cells evaluated by our novel method for protein density analysis (see also Supplementary Figure 9). Expression levels were determined by summing up the intensity across the raw sequence of TIRF images. Median intensity was calculated in the ROI of tested samples (the same ROI as we used for the SOFI-based clustering analysis). The results reveal comparable expression level of tested CD4 variants. In each box plot, the box represents the interquartile range (IQR), the central mark is the median, and the whiskers extend to the most extreme data points.

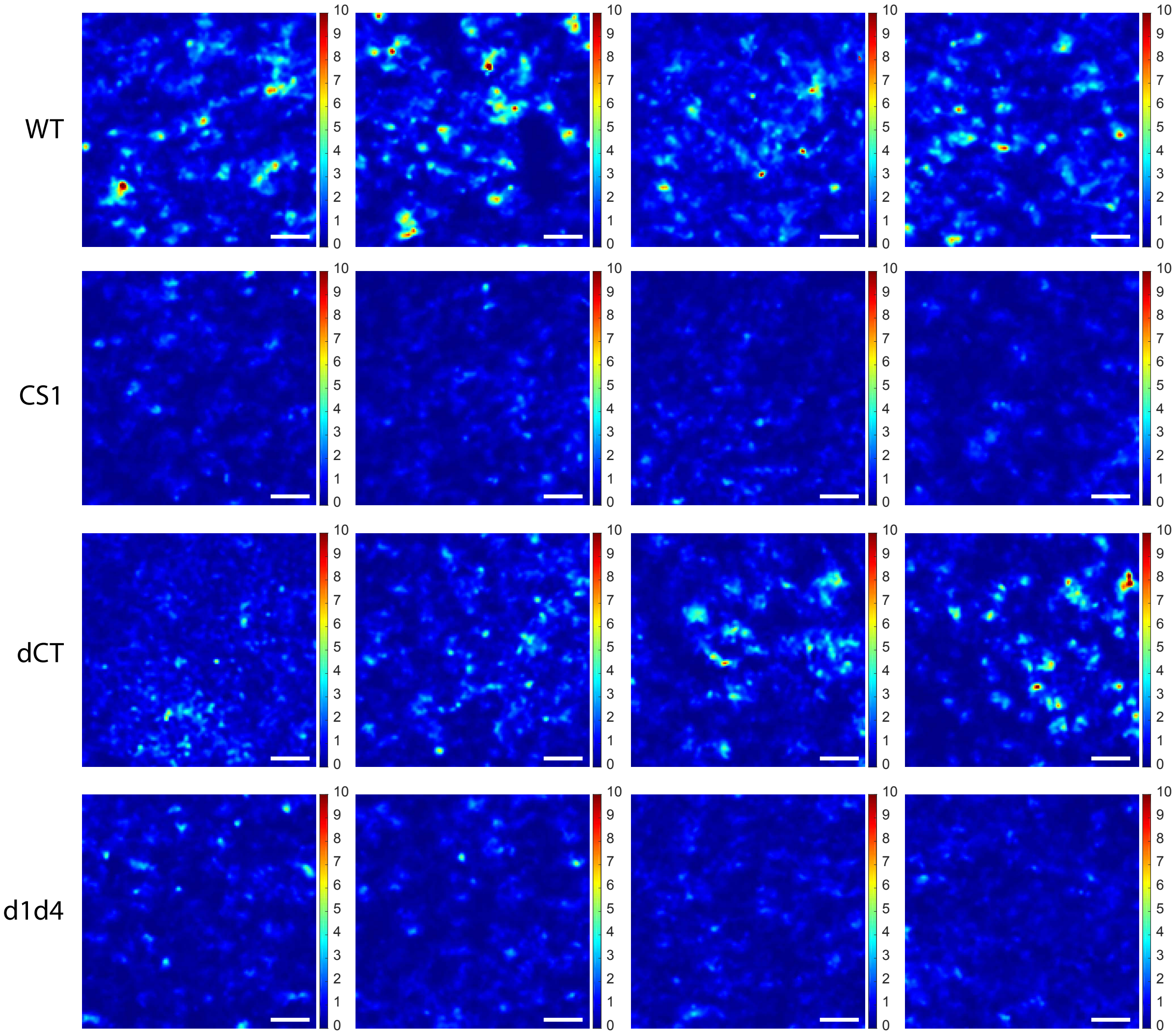

Supplementary Figure 9: SOFI molecular density maps of CD4 variants expressed as mEos2 fusion proteins in resting T cells immobilised on poly-L-lysine coated glass coverslips. Cells were imaged using TIRF illumination and the acquired data were analyzed using our bSOFI algorithm (see Methods). Each row represents four randomly selected ROIs/samples for each studied CD4 variant. Colorbars represent relative density ($\#\delta_{avg}$; see Supplementary Fig. 1). Scale bar 500 nm. Presented molecular density maps demonstrate differences in the plasma membrane distribution of CD4 variants. CD4 WT exhibits numerous large, dense and sometimes interconnected HDRs. On the contrary, non-palmitoylatable CD4 mutant (CS1) and the variant lacking the extracellular domain (dD1D4) are randomly distributed with only a few small HDRs detected. The CD4 variant lacking the cytoplasmic tail (dCT) represents an intermediate phenotype. Cells exhibiting the protein accumulation in the large and dense HDRs were found (see the two images on the right), but the majority of cases was in rather small HDRs with medium protein density (see the two images on the left). These data indicate that the extracellular domain and palmitoylation are essential for the native distribution of CD4 on resting T cells.

Supplementary Note 1

The technical requirements for SOFI are a classical widefield microscope merged with a fast high sensitivity digital camera. SOFI image processing is based on higher order statistics and exploits the temporal sequence of blinking fluorescent emitters [4, 5]. Calculating spatio-temporal cross-cumulants allows SOFI to obtain a super-resolved, background-free and noise-reduced images. Higher-order cumulants contain information about the photo-physics of the emitters. Combining SOFI images of different cumulant orders, allows one to extract physical parameters like molecular density [6], which we applied to investigate plasma membrane distribution of proteins.

SOFI principle and theory

As stated by Dertinger et al. [4], the fluctuating emitters should switch between at least two optically distinguishable states (e.g. a dark and a bright state) repeatedly and independently in a stochastic manner. Images of stochastically blinking emitters are recorded such that the point-spread function (PSF) extends over several camera pixels. Acquiring a sequence of images results in a time dependent intensity trace for each pixel. Assuming N independently fluctuating emitters, the detected intensity is given as

$$I(\mathbf{r},t) = \sum_{k=1}^{N} \epsilon_k U(\mathbf{r}-\mathbf{r}_k) s_k(t) + b(\mathbf{r}) + n(\mathbf{r},t), \tag{1}$$

where ϵ_k is the molecular brightness, $U(\mathbf{r}-\mathbf{r}_k)$ is the PSF at the position $\mathbf{r}_k$, $s_k(t)$ denotes a switching function (normalized fluctuation sequence, $s_k(t) \in \{0,1\}$), $b(\mathbf{r})$ is a constant background, and $n(\mathbf{r},t)$ represents an additive noise contribution.

For each pixel, an nth order cumulant is calculated for disentangling emitters inside the PSF. By applying the nth order cumulant to Eq. (1), we obtain

$$\kappa_n\{I(\mathbf{r},t)\}(\tau) = \kappa_n \left\{ \sum_{k=1}^{M} \epsilon_k U(\mathbf{r}-\mathbf{r}_k) s_k(t) + b(\mathbf{r}) + n(\mathbf{r},t) \right\} (\tau). \tag{2}$$

Using additivity and semi-invariance properties of cumulants [7], the nth order cumulant with zero time lag can be written as

$$\kappa_n\{I(\mathbf{r},t)\} = \sum_{k=1}^{N} \epsilon_k^n U^n(\mathbf{r}-\mathbf{r}_k) \kappa_n\{s_k(t)\} + \kappa_n\{b(\mathbf{r})\} + \kappa_n\{n(\mathbf{r},t)\}. \tag{3}$$

For $(n \geq 2)$, the Gaussian noise $(\kappa\{n(\mathbf{r},t)\})$ and stationary background $(\kappa\{b(\mathbf{r})\}$) terms are eliminated by the cumulant analysis as an intrinsic property of cumulants. For an nth order cumulant, the PSF is raised to the nth power (see Eq. 3). As a consequence, the PSF is narrowed and the spatial resolution is improved by a factor of $\sqrt{n}$ [4]. Therefore, increasing the cumulant order yields an image with an enhanced spatial resolution. Since a multiplication in the spatial domain corresponds to a convolution in the frequency domain, the cut-off frequency of the spectrum $\tilde{U}^n(\mathbf{k})$ is n-times higher than that of $\tilde{U}(\mathbf{k})$. By applying a deconvolution and a subsequent rescaling, the nth order cumulant image exhibits an up to n-fold resolution improvement [5]. As shown in [5], virtual pixels can be calculated in between the physical pixels using cross-cumulants and followed by a flattening operation i.e. assigning proper weights to these virtual pixels [5, 8, 9].

SOFI assumes a blinking model where the fluorophores reversibly switch between a bright and a dark state. In Deschout et al. [10], SOFI was applied to the PALM photo-physical model. In the PALM photo-physical model, the emitter activation is assumed as non-reversible, however, once the emitter is activated, it exhibits several fast blinking events prior to the final

bleaching event [11]. The emitter fluctuates between two different states (an on-state S_{on} and a dark state S_{off}), which is expressed by the on-time ratio as

$$\rho = \frac{\tau_{\text{on}}}{\tau_{\text{on}} + \tau_{\text{off}}}, \tag{4}$$

where τ_{on} and τ_{off} are the characteristic lifetimes of the S_{on} and S_{off} states. The nth order cumulant $\kappa_n\{s_k(t)\}$ is in this model described by a Bernoulli distribution with probability ρ_{on} [6] and approximated by an nth order polynomial function for the on-time ratio as

$$f_n(\rho_{\text{on}}) = \rho_{\text{on}}(1-\rho_{\text{on}})\frac{\partial f_{n-1}}{\partial \rho_{\text{on}}}. \tag{5}$$

Under these conditions, the nth order cumulant can be approximated as [6]

$$\kappa_n\{I(\mathbf{r},\mathbf{t})\} \approx \epsilon^n f_n(\rho_{\text{on}}) \sum_{k=1}^{N} U^n(\mathbf{r}-\mathbf{r}_k). \tag{6}$$

Estimation of density maps

Geissbuehler et al. [6] used three cumulant images (2nd, 3rd, and 4th order) to estimate molecular parameters: on-time ratio, brightness and molecular density. Here, we generalize this concept to any three cumulant images of distinct orders. If we assume spatially varying but locally constant on-time ratios and molecular brightness, the cumulants (for the cumulant order $n > 1$) can be approximated by [6]

$$g_n(\mathbf{r}) \approx \epsilon^n(\mathbf{r}) f_n(\rho_{\text{on}}) N(\mathbf{r}) \mathcal{E}_V\{U^n(\mathbf{r})\}. \tag{7}$$

where $\mathcal{E}_V\{U^n(\mathbf{r})\}$ is the expectation value of $U^n(\mathbf{r})$, $N(\mathbf{r})$ is the number of emitters inside a detection volume V. Approximating the PSF near the interface in a total internal reflection (TIR) configuration by a lateral 2D Gaussian profile combined with an axial exponential profile, we obtain

$$\mathcal{E}_V\{U^n_{\text{TIR}}(\mathbf{r})\} = \frac{c(\sigma_{x,y}, \sigma_z, d_z)}{n^2}, \tag{8}$$

where d_z represents the exponential decay of the TIR illumination [12].

Using 3 consecutive cumulant images of orders $n, (n-1), (n-2)$, we obtain for the ratios

$$K_1 = \frac{g_{n-1}}{g_{n-2}} = \frac{f_{n-1}(\rho_{\text{on}}(\mathbf{r}))}{f_{n-2}(\rho_{\text{on}}(\mathbf{r}))}\epsilon(\mathbf{r})\frac{\mu_{n-1}}{\mu_{n-2}} \tag{9}$$

$$K_2 = \frac{g_n}{g_{n-2}} = \frac{f_n(\rho_{\text{on}}(\mathbf{r}))}{f_{n-2}(\rho_{\text{on}}(\mathbf{r}))}\epsilon^2(\mathbf{r})\frac{\mu_n}{\mu_{n-2}} \tag{10}$$

$$K_3 = \frac{g_n}{g_{n-1}} = \frac{f_n(\rho_{\text{on}}(\mathbf{r}))}{f_{n-1}(\rho_{\text{on}}(\mathbf{r}))}\epsilon(\mathbf{r})\frac{\mu_n}{\mu_{n-1}}, \tag{11}$$

where $\mu_n = \mathcal{E}_V\{U^n(\mathbf{r})\}$. Substitution of Eq. (11) into Eq. (9) leads to

$$\frac{g_n g_{n-2}}{g^2_{n-1}} = \frac{f_n(\rho_{\text{on}}(\mathbf{r})) f_{n-2}(\rho_{\text{on}}(\mathbf{r}))}{f^2_{n-1}(\rho_{\text{on}}(\mathbf{r}))}. \tag{12}$$

$$\left\{\epsilon(\mathbf{r}) = \frac{g_n}{g_{n-1}}\frac{f_3(\rho_{\text{on}}(\mathbf{r}))}{f_n(\rho_{\text{on}}(\mathbf{r}))}\frac{\mu_{n-1}}{\mu_n}, N(\mathbf{r}) = \frac{g_n(\mathbf{r})}{\epsilon^n(\mathbf{r}) f_n(\rho_{\text{on}})\mu_n}\right\}. \tag{13}$$

Building up the ratios K_1 and K_2 from the Eq. (14) for cumulants of $2^{nd}, 3^{rd}$, and 4th order, we obtain

$$K_1(\mathbf{r}) = \frac{\mu_2 g_3}{\mu_3 g_2}(\mathbf{r}) = \epsilon(\mathbf{r})(1 - 2\rho_{\text{on}}(\mathbf{r})) \tag{14}$$

$$K_2(\mathbf{r}) = \frac{\mu_2 g_4}{\mu_4 g_2}(\mathbf{r}) = \epsilon^2(\mathbf{r})(1 - 6\rho_{\text{on}}(\mathbf{r}) + 6\rho_{\text{on}}^2(\mathbf{r})), \tag{15}$$

Solving for molecular brightness ϵ, on-time ratio ρ_{on}, we obtain two solutions for the on-time ratio ρ_{on}, and molecular brightness ϵ

$$\left\{ \rho_{\text{on}}(\mathbf{r}) = \frac{3K_1^2 \pm K_1\sqrt{3K_1^2 - 2K_2} - 2K_2}{2(3K_1^2 - 2K_2)}, \epsilon(\mathbf{r}) = \mp\sqrt{3K_1^2 - 2K_2} \right\}, \tag{16}$$

where the first solution corresponds to a negative brightness and will be discarded. The molecular density (number of molecules N per pixel area) is

$$N(\mathbf{r}) = \frac{g_2(\mathbf{r})}{\epsilon^2(\mathbf{r})\rho_{\text{on}(\mathbf{r})}(1 - \rho_{\text{on}}(\mathbf{r}))}. \tag{17}$$

For cumulants of $3^{rd}, 4^{th}$, and 5th order, the ratios K_1 and K_2 become

$$K_1(\mathbf{r}) = \frac{\epsilon(\mathbf{r})(1 - 6\rho_{\text{on}} + 6\rho_{\text{on}}^2)}{(1 - 2\rho_{\text{on}}(\mathbf{r}))} \tag{18}$$

$$K_2(\mathbf{r}) = \epsilon^2(\mathbf{r})(12\rho_{\text{on}}^2 - 12\rho_{\text{on}} + 1) \tag{19}$$

which ends in four solutions. Two correspond to positive molecular brightness

$$\rho_{\text{on}}(\mathbf{r})_{1,2} = \frac{12K_1^3 \pm \sqrt{3}\sqrt{K_1^2(4K_1^2 - 3K_2)}\sqrt{4K_1^2 \mp 2\sqrt{K_1^2(4K_1^2 - 3K_2)} - 3K_2} - 9K_1K_2}{6K_1(4K_1^2 - 3K_2)}, \tag{20}$$

$$\epsilon(\mathbf{r})_{1,2} = \frac{\sqrt{4K_1^2 \mp 2\sqrt{K_1^2(4K_1^2 - 3K_2)} - 3K_2}}{\sqrt{3}}. \tag{21}$$

Using a combination of higher order cumulants for molecular parameters can theoretically provide higher spatial resolution of the molecular parameter maps assuming high enough SNR of the cumulant images used. For the combination of $4^{th}, 5^{th}, 6^{th}$ order cumulant, it is also possible to find a solution in a closed form, but due to its complexity, a numerical approach might be preferred.

Therefore SOFI extracts density without counting individual events in the image. Density simply results from a correlation/cumulant analysis of intensity time traces.

Supplementary References

[1] I. Chamma, F. Levet, J.-B. Sibarita, M. Sainlos, and O. Thoumine, "Nanoscale organization of synaptic adhesion proteins revealed by single-molecule localization microscopy," *Neurophotonics*, vol. 3, p. 041810, 2016.

[2] A. Burgert, S. Letschert, S. Doose, and M. Sauer, "Artifacts in single-molecule localization microscopy," *Histochemistry and Cell Biology*, vol. 144, pp. 123–131, 2015.

[3] F. Levet, E. Hosy, A. Kechkar, C. Butler, A. Beghin, D. Choquet, and J.-B. Sibarita, "SR-Tesseler: a method to segment and quantify localization-based super-resolution microscopy data.," *Nature methods*, vol. 12, pp. 1065–71, 2015.

[4] T. Dertinger, R. Colyer, G. Iyer, S. Weiss, and J. Enderlein, "Fast, background-free, 3D super-resolution optical fluctuation imaging (SOFI).," *Proceedings of the National Academy of Sciences of the United States of America*, vol. 106, no. 52, pp. 22287–92, 2009.

[5] T. Dertinger, R. Colyer, R. Vogel, J. Enderlein, and S. Weiss, "Achieving increased resolution and more pixels with Superresolution Optical Fluctuation Imaging (SOFI).," *Optics express*, vol. 18, pp. 18875–85, Aug. 2010.

[6] S. Geissbuehler, N. L. Bocchio, C. Dellagiacoma, C. Berclaz, M. Leutenegger, and T. Lasser, "Mapping molecular statistics with balanced super-resolution optical fluctuation imaging (bSOFI)," *Optical Nanoscopy*, vol. 1, no. 1, 2012.

[7] J. M. Mendel, "Tutorial on Higher-Order Statistics (Spectra) in Signal Processing and System Theory: Theoretical Results and Some Applications," *Proceedings of the IEEE*, vol. 79, pp. 278–305, 1991.

[8] S. C. Stein, A. Huss, D. Hähnel, I. Gregor, and J. Enderlein, "Fourier interpolation stochastic optical fluctuation imaging.," *Optics express*, vol. 23, pp. 16154–63, 2015.

[9] W. Vandenberg, S. Duwé, M. Leutenegger, B. Krajnik, T. Lasser, and P. Dedecker, "Model-free uncertainty estimation in Stochastical Optical Fluctuation Imaging (SOFI) leads to a doubled temporal resolution," vol. 2402, pp. 1347–1355, 2015.

[10] H. Deschout, T. Lukes, A. Sharipov, D. Szlag, L. Feletti, W. Vandenberg, P. Dedecker, J. Hofkens, M. Leutenegger, T. Lasser, and A. Radenovic, "Complementarity of PALM and SOFI for super-resolution live-cell imaging of focal adhesions," *Nature Communications*, vol. 7, p. 13693, 2016.

[11] N. Durisic, L. Laparra-Cuervo, A. Sandoval-Álvarez, J. S. Borbely, and M. Lakadamyali, "Single-molecule evaluation of fluorescent protein photoactivation efficiency using an in vivo nanotemplate.," *Nature methods*, vol. 11, pp. 156–62, 2014.

[12] K. Hassler, T. Anhut, R. Rigler, M. Gösch, and T. Lasser, "High Count Rates with Total Internal Reflection Fluorescence Correlation Spectroscopy," *Biophysical journal*, vol. 88, pp. L01–L03, 2005.
